# Site-Specific Reprogramming of Macrophage Responsiveness to Bacterial Lipopolysaccharide in Obesity

**DOI:** 10.3389/fimmu.2019.01496

**Published:** 2019-06-28

**Authors:** Evilin N. Komegae, Monique T. Fonseca, Sanseray da Silveira Cruz-Machado, Walter M. Turato, Luciano R. Filgueiras, Regina P. Markus, Alexandre A. Steiner

**Affiliations:** ^1^Neuroimmunology of Sepsis Laboratory, Department of Immunology, Institute of Biomedical Sciences, University of São Paulo, São Paulo, Brazil; ^2^Laboratory of Chronopharmacology, Institute of Biosciences, University of São Paulo, São Paulo, Brazil; ^3^Department of Clinical and Toxicological Analyses, School of Pharmaceutical Sciences, University of São Paulo, São Paulo, Brazil

**Keywords:** obese, fat, diet, immune, inflammation, host defense, LPS, cytokine

## Abstract

The mechanisms by which obesity may alter immune responses to pathogens are poorly understood. The present study assessed whether the intrinsic responsiveness of resident macrophages to bacterial lipopolysaccharide (LPS) is reprogrammed in high-fat diet (HFD)-induced obesity. Macrophages from adipose tissue, lung alveoli, and the peritoneal cavity were extracted from obese rats on a HFD or from their lean counterparts, and subsequently studied in culture under identical conditions. CD45^+^/CD68^+^ cells (macrophages) were abundant in all cultures, and became the main producers of TNF-α upon LPS stimulation. But although all macrophage subpopulations responded to LPS with an M1-like profile of cytokine secretion, the TNF-α/IL-10 ratio was the lowest in adipose tissue macrophages, the highest in alveolar macrophages, and intermediary in peritoneal macrophages. What is more, diet exerted qualitatively distinct effects on the cytokine responses to LPS, with obesity switching adipose tissue macrophages to a more pro-inflammatory program and peritoneal macrophages to a less pro-inflammatory program, while not affecting alveolar macrophages. Such reprogramming was not associated with changes in the inflammasome-dependent secretion of IL-1β. The study further shows that the effects of diet on TNF-α/IL-10 ratios were linked to distinct patterns of NF-κB accumulation in the nucleus: while RelA was the NF-κB subunit most impacted by obesity in adipose tissue macrophages, cRel was the subunit affected in peritoneal macrophages. It is concluded that obesity causes dissimilar, site-specific changes in the responsiveness of resident macrophages to bacterial LPS. Such plasticity opens new avenues of investigation into the mechanisms linking obesity to pathogen-induced immune responses.

## Introduction

The biology of macrophages in obesity has been extensively studied from the perspective of the low-grade sterile inflammation that drives adipose tissue remodeling and insulin resistance; for a review, see Lumeng and Saltiel (1). However, it is currently unclear whether and how obesity also affects the acute responses of macrophages to pathogens or pathogen-associated molecular patterns (PAMPs). This question is relevant in view of the evidence indicating that obesity impacts the outcome of infectious diseases in humans (2, 3), and experimental animals (4–6), with both favorable and unfavorable outcomes having been reported.

Pathogen-induced immune responses differ from low-grade sterile inflammation for being phasic and substantially more pronounced. To grasp the magnitude of this difference, one should consider that while the steady-state level of TNF-α in the plasma of uninfected obese mice rarely exceeds 20 pg/ml (7, 8), it usually peaks at more than 1,000 pg/ml within 2 h of a challenge with bacterial lipopolysaccharide (LPS) or within 8 h of septic peritonitis (9, 10). Accordingly, although there are reports of adipose tissue macrophages sometimes switching their resting state in obesity from an anti-inflammatory, M2-like profile to a pro-inflammatory profile in the lower end of the M1-like range (11–14), it is presently unknown if a similar shift in macrophage polarity could occur for PAMP-induced responses in the upper end of the M1-like range. Furthermore, as far as macrophage responses to pathogens or PAMPs are concerned, the link between obesity and immune responses ought to be investigated not only in adipose tissue, but also in sites that are at the front line of host defense, such as the lungs and peritoneum. And in view of the reported heterogeneity of macrophage subpopulations (15, 16), site-specific response patterns can be expected.

Another point that deserves investigation is whether the influence of obesity on PAMP-induced responses relies on extrinsic factors that influence macrophages in the tissue microenvironment or on longer-lasting reprogramming of intrinsic macrophage properties (e.g., via epigenetic mechanisms). One way to dissect out these factors is to extract macrophages from the distinct tissue microenvironments of obese vs. lean animals, so that they can be subsequently studied *ex vivo* under identical conditions. Using this sort of approach, we have observed that even a brief change in energy balance (24 h of food deprivation) is able to reprogram the intrinsic responsiveness to LPS in certain macrophage subpopulations (17). Thus, prominent changes in the intrinsic program of macrophages to LPS can be expected in obese animals chronically maintained on a high-fat diet (HFD).

Here, to probe for site-specific effects of obesity on the intrinsic responsiveness of macrophages to a PAMP, resident macrophages were harvested from obese vs. lean rats, and subsequently stimulated with bacterial LPS under identical culture conditions. Three macrophage subpopulations were studied: macrophages of the adipose tissue stromal vascular fraction (AT-SVF); alveolar (lung) macrophages; and peritoneal macrophages. Macrophage responsiveness was assessed based on the secretion of early pro-inflammatory cytokines (TNF-α and IL-1β), and, as a counterpoint, on the secretion of an anti-inflammatory cytokine (IL-10). The rat was chosen as the model species, because it was large enough to provide sufficient numbers of resident macrophages for cultures that yielded detectable levels of cytokines in medium supernatants. We chose not to use eliciting agents such as thioglycolate to enrich the sites of interest with macrophages, because such enrichment involves infiltrating, monocyte-derived macrophages that are known to be phenotypically distinct from resident macrophages (18, 19).

## Materials and Methods

### Animals and Diet-Induced Obesity

Male Wistar rats were obtained from the specific pathogen-free (SPF) animal facility of the University of São Paulo. From the day of weaning (21 days after birth), rats with free access to water were given a HFD (60% of calories from fat, 5.1 kcal/g; Rhoster, Araçoiaba da Serra, SP, Brazil) or a low-fat diet (LFD: 12% of calories from fat, 3.7 kcal/g; Nuvilab, Curitiba, PR, Brazil). The diets were similar in terms of protein content (23% in HFD and 22% in LFD), and both of them conformed to SPF standards. The rats were caged in groups of three or four. The rats were weighed on a daily basis. Caloric intake was also estimated on a daily basis, in this case by multiplying the mass of food consumed in a cage by the appropriate caloric content of the diet (5.1 or 3.7 kcal/g, see above), and then dividing this product by the number of rats in the corresponding cage. The animal room was on a 12:12 h light-dark cycle (lights on at 7 a.m.). With the aim of providing a thermally comfortable environment to the grouped rats (20), room temperature was maintained at 24-27°C. On the 21st week post-weaning, body fat content was estimated by dual-energy X-ray absorptiometry scan, as described elsewhere (21). Between post-weaning weeks 24 and 28, the rats were anesthetized with isoflurane (1.5-2.5%) for harvesting of tissue macrophages. All protocols were approved by the Animal Care and Use Committee at the Institute of Biomedical Sciences of the University of São Paulo.

### AT-SVF Macrophages

Epididymal adipose tissue was dissociated with type-II collagenase (Sigma C6885; 2 mg/ml) in an incubator orbital shaker (40 min, 37°C). The collagenase was then inactivated by dilution with HBSS (Hank's balanced salt solution) containing 2% FBS (fetal bovine serum), and the cell extract was centrifuged at 300 × *g* for 10 min at 25°C. This centrifugal force was sufficient to pull down macrophage-enriched AT-SVF cells, but not fat-loaded (less dense) adipocytes. The pelleted cells were resuspended and incubated with ACK lysis buffer (ThermoFisher A1049201) for 3 min at room temperature to eliminate erythrocytes. Next, the cells were washed with HBSS supplemented with 2% FBS, and passed through a 100 μm-mesh cell strainer. After the cells were pelleted by another round of centrifugation at 300 × *g* (10 min, 25°C), they were resuspended in HEPES-containing RPMI 1640 GlutaMAX medium (ThermoFisher 72400-047) supplemented with penicillin (100 U/ml), streptomycin (100 μg/ml) and FBS (10%). The AT-SVF cell extract was cultured in this medium at 37°C and 5% CO_2_ at a density of 1 × 10^6^ live cells (trypan blue exclusion) per well in 12-well, flat-bottom polystyrene plates. Because adherence of the AT-SVF macrophages to the cultureware was weak, adherent and non-adherent cells could not be separated in this preparation. The cells were allowed to rest in culture for 1 h before being stimulated with LPS (see *Macrophage Stimulation*, below).

### Alveolar and Peritoneal Macrophages

Alveolar and peritoneal cells were harvested by lavage of the corresponding sites with ice-cold PBS (phosphate-buffered saline). The alveolar lavage was performed in two rounds, each with 60 ml of PBS, whereas the peritoneal lavage was performed in a single round with 60 ml of PBS. The cell extract was concentrated by centrifugation (600 × *g*, 10 min, 4°C), and the pellet was resuspended and incubated with ACK lysis buffer for 3 min at room temperature. The resulting cell extract was then pelleted by centrifugation, and resuspended in HEPES-containing RPMI 1640 GlutaMAX medium supplemented with penicillin (100 U/ml), streptomycin (100 μg/ml) and FBS (2 or 10%, see below). The cells were then plated at a density of 1 × 10^6^ live cells (trypan blue exclusion) per well in 12-well, flat-bottom polystyrene plates. The cells were cultured at 37°C and 5% CO_2_. Non-adherent cells were discarded twice: the first time took place 30 min after incubation with medium containing 2% FBS; the second time took place after an overnight incubation with medium containing 10% FBS. Subsequently, the adhered cells were studied in medium containing 10% FBS. A 1-h resting period was allowed between the second medium change and the stimulation with LPS (see *Macrophage Stimulation*, below).

### Macrophage Stimulation

Cells were stimulated with LPS from *E. coli* O127:B8 (Sigma L3129), added to the culture medium at the volume of 10 μl/ml to a final concentration of 500 ng/ml. Control, unstimulated cultures received the same volume of pyrogen-free sterile saline. The duration of LPS stimulation varied depending on the endpoint of interest. A 4-h stimulation period was used for cytokine determination in culture supernatants. A 2-h period was used to assess the co-localization of macrophage markers with TNF-α in the cell fraction. And a 10-min period was employed when the endpoint of interest was the nuclear accumulation of NF-κB.

### Quantification of Secreted Cytokines

TNF-α, IL-1β, and IL-10 were measured in cell-culture supernatants by sandwich ELISA using reagents from R&D Systems (cat. Nos. DY510, DY501, and DY522, respectively), according to the manufacturer's instructions. Detection ranges were 16-2,000 pg/ml for all cytokines assayed (*r*^2^ > 0.97; four-parameter logistic regression).

### Cytometry-Based Analysis of Macrophage Markers and TNF-α

Adherent cells were detached from the cultureware by incubation (10 min) in ice-cold PBS, followed by scrapping with a syringe barrel. The cells were then transferred to cytometry tubes (5 × 10^5^ cells/tube), where they were subjected to the following sequential steps: (i) blockade with BD's Stain Buffer (cat. No. 554656; 30 min, 4°C); (ii) surface staining for CD45 (a leukocyte marker); (iii) permeabilization with BD's Phosflow Perm Buffer III (cat. No. 558050; 20 min, 4°C, in the dark); and (iv) intracellular staining for CD68/ED1 (a macrophage marker) and TNF-α. Stainings were achieved by 30-min incubation with the following monoclonal antibodies at 4°C and in the dark: mouse anti-rat CD45 conjugated with APC-Cy7 (BD 561586; 1:100); mouse anti-rat CD68 conjugated with PE (Novus Biologicals NB600-985PE; 1:100); and hamster anti-rat/mouse TNF-α conjugated with APC (BioLegend 506107; 1:25). After washing with cold PBS, stained and unstained cells were passed through a FACSCANTO II flow cytometer (BD Biosciences). Overlapping regions in fluorochrome spectra were compensated prior to data acquisition in the FACSDiva software. Post-acquisition analysis of the data was performed in the FlowJo software.

### Nuclear Accumulation of NF-κB

The accumulation of NF-κB subunits in the nucleus was assessed by imaging flow cytometry in an AMNIS Flowsight System (AMNIS Corporation). After cells were detached from the cultureware as described above and fixed with Biolegend's Fixation Buffer (cat. No. 420801), they were permeabilized with saponin 0.5% at room temperature for 10 min. All subsequent incubations and stainings were performed at 4°C, in the dark. Non-specific binding sites were blocked by a 1-h incubation with BSA (bovine serum albumin, 3%), donkey normal serum (3%), and glycine (0.3 M). Intracellular staining for CD68 was achieved by incubation with an unconjungated monoclonal mouse antibody (abcam AB31630; 1:200, 1 h), which was revealed by a secondary, FITC-conjugated donkey anti-mouse antibody (Invitrogen A24501; 1:200, 1 h). Intracellular staining for each known NF-κB subunit was achieved by overnight incubation with unconjugated rabbit polyclonal antibodies against RelA/p65 (abcam AB7970; 1:200), RelB (Santa Cruz sc-226; 1:200), cRel (Santa Cruz sc-70; 1:500), p50/p105 (abcam AB7971; 1:200) or p52/p100 (abcam AB7972; 1:200). The primary anti-NF-κB antibodies were revealed using a PE-conjugated donkey anti-rabbit polyclonal antibody (Biolegend 406421; 1:200, 1 h). For nuclear localization, the cells were also stained with propidium iodide (PI; Sigma P4170; 1:1,000) immediately before analysis. Negative controls were incubated with the secondary antibodies in the absence of any primary antibody. The specificity of the antibodies used has been validated in a previous study (22).

Data analysis was performed using the Ideas software (AMNIS Corporation), according to the following workflow: (i) a compensation matrix for spillover across fluorochrome spectra was applied to all events, (ii) compensated events were sequentially gated for focus, singlets and CD68^+^/PI^+^, (iii) a nuclear mask was created at the threshold for PI staining, and (iv) fluorescence intensity for NF-κB staining was determined within the nuclear mask.

### Data Processing and Analyses

To evaluate cytokine secretion relative to the number of cultured macrophages, the following equation was used: cytokine secreted per CD45^+^/CD68^+^ cell = (cytokine concentration × volume of medium)/(number of cells recovered from the culture × relative abundance of CD45^+^/CD68^+^ cells). This analysis was based on the premise that macrophages are the major producers of early cytokines in these cultures, which was verified experimentally (see *Results*). The ratios of secreted TNF-α and IL-1β to secreted IL-10 were used as indices of the balance between pro-inflammatory and anti-inflammatory signals. Nuclear accumulation of NF-κB subunits in LPS-stimulated macrophages was expressed as HFD-induced fold change in relation to the LFD group.

Statistical comparisons were performed using Statistica 8.0 (StatSoft). After normality was confirmed by the Kolmogorov-Smirnov test, the data were evaluated by parametric tests with the level of significance at *p* < 0.05. ANOVA, followed by the Fisher LSD test was used to assess how diet and LPS stimulation affected cytokine secretion. Repeated-measures ANOVA (also followed by the Fisher LSD test) was employed to compare caloric intake and body mass across time points and diet. Pairwise *t*-test was used to evaluate the effect of diet on cytokine ratios or fat mass. Lastly, HFD-induced fold changes in the nuclear content of NF-κB subunits were evaluated by single-sample *t*-test, with the null hypothesis set to fold change of one (i.e., no change).

## Results

### Diet-Induced Obesity

**Figure 1:**
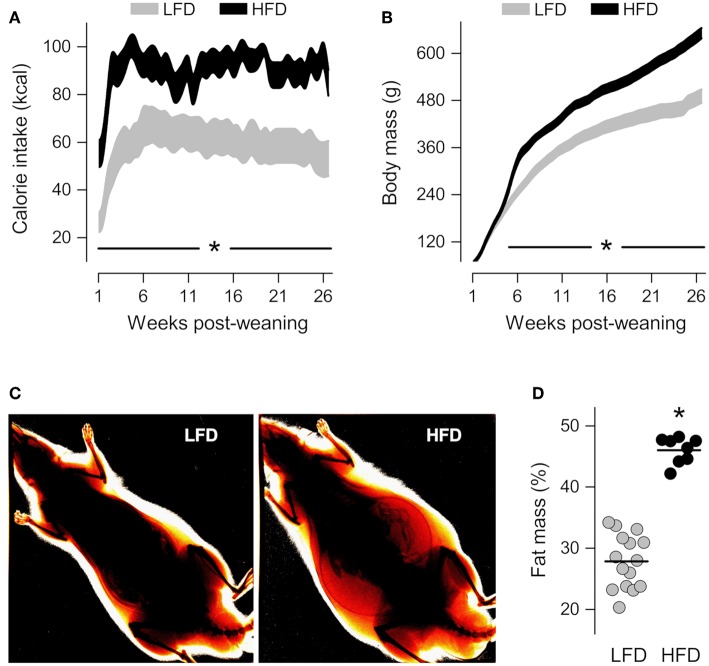
Characterization of diet-induced obesity. **(A,B)** Time-courses of calorie intake and body mass from the first day post-weaning, when the rats began to feed on a low-fat diet (LFD) or a high-fat diet (HFD). The data are shown as 95% confidence intervals. The number of animals was 12 in the LFD group and 16 in the HFD group. **(C)** Representative ratiometric X-ray images thresholded to highlight peripheral fat of LFD and HFD rats at the 21st week post-weaning. **(D)** Relative fat mass derived from the analysis of ratiometric X-ray images. In **(D)**, each symbol denotes an individual value; the horizontal line indicates the group means. *Statistical difference (*p* < 0.05) between the LFD and HFD groups.

Compared to their LFD-fed counterparts, the HFD-fed rats exhibited a higher caloric intake from the first day post-weaning (Figure 1A), but this higher intake did not immediately impact body mass (Figure 1B). It took 4 weeks for the body mass of the HFD-fed rats to become significantly higher than that of the LFD-fed rats (Figure 1B), but, after that point, the intergroup difference in body mass increased progressively. On the 21st week post-weaning, dual-energy X-ray absorptiometry scans revealed that animals of the HFD group had 65% more body fat than their LFD-group counterparts (Figures 1C,D). And since there was no overlap in body fat content between the HFD and LFD groups (Figure 1D), it was considered that obesity was fully established from that point onward. Between post-weaning weeks 24 and 28, resident macrophages were extracted from their distinct tissue microenvironments in obese vs. lean animals, and studied in culture under identical conditions.

### AT-SVF Macrophages

**Figure 2:**
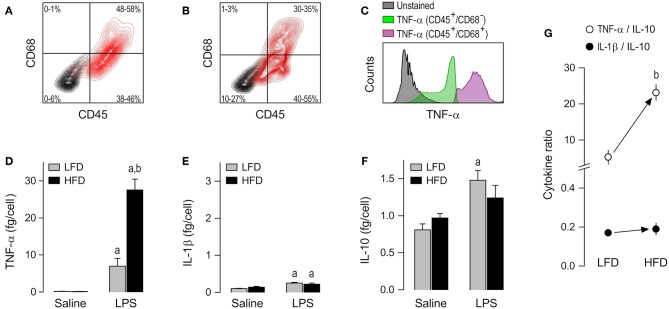
Macrophage abundance and cytokine secretion in AT-SVF cultures. **(A,B)** Representative flow-cytometry density plots of staining for CD45 (a leukocyte marker) and CD68 (a rat macrophage marker) in AT-SVF cells from LFD-fed rats **(A)** or HFD-fed rats **(B)**. Stained cells are shown in red; unstained cells are shown in gray. Group results—*n* = 5 culture replicates in **(A)**; *n* = 12 in **(B)**—are shown as 95% confidence intervals within each quadrant. **(C)** Representative histograms showing that LPS-stimulated cells that are CD45^+^/CD68^+^ (macrophages) stain more strongly for TNF-α than the other cellular phenotypes present in the AT-SVF culture. For staining intensities of all samples, see Supplemental Table 1. **(D–F)** Effects of diet and *ex-vivo* stimulation with LPS on the secretion of TNF-α **(D)**, IL-1β **(E)** and IL-10 **(F)** by AT-SVF cells. To aid comparison of this macrophage subpopulation with the others, the scales in these panels are identical to those of the corresponding panels in Figures 3, 4. Data in **(D–F)** are expressed as means ± SEM. Sample size (number of culture replicates) was 4 in the LFD group not stimulated with LPS (saline), 6 in the HFD group not stimulated with LPS (saline), 5 in the LFD group stimulated with LPS, and 7 in the HFD group stimulated with LPS. **(G)** TNF-α/IL-10 and IL-1β/IL-10 ratios (means ± SEM) in the LPS-stimulated AT-SVF cultures from the LFD vs. HFD groups. Statistical marks: ^a^significant effect of LPS (compared to saline), ^b^significant effect of diet.

The presence of macrophages in AT-SVF cultures was confirmed by co-staining for CD45 [a leukocyte common antigen (23)] and CD68 [a well-validated macrophage marker in the rat (24)]. CD45^+^/CD68^+^ cells made up 48–58% of the AT-SVF cultures from LFD-fed rats (Figure 2A) and 30–35% of the AT-SVF cultures from HFD-fed rats (Figure 2B). The lower yield of macrophages in the latter group seems to have resulted from the formation of more macrophage-adipocyte aggregates in the cell fraction discarded during the purification process. In both groups, though, CD45^+^/CD68^+^ cells (macrophages) were the main producers of TNF-α (Figure 2C and Supplemental Table 1).

Having validated the experimental preparation, we moved on to comparing cytokine secretion in AT-SVF cultures derived from HFD- vs. LFD-fed rats. To account for inter-group differences in the abundance of cultured macrophages, cytokine content in culture medium was expressed as a ratio to the number of CD45^+^/CD68^+^ cells, as detailed in *Data Processing and Analyses* (*Materials and Methods*). No detectable effect of diet was found on the basal release of TNF-α, IL-1β or IL-10 by AT-SVF cells not stimulated with LPS. However, a major effect of diet was revealed on LPS-induced TNF-α secretion. This was the cytokine most affected by LPS in AT-SVF cultures, and its secretion in this condition was four times more pronounced in the HFD group than in the LFD group (Figure 2D). LPS also exerted statistically significant effects on the secretion of IL-1β (Figure 2E), but this effect was minor and not affected by diet. Regarding IL-10, the experiments revealed that while the basal secretion of this cytokine was relatively high in AT-SVF cultures, the effect of LPS on its secretion was rather minor (Figure 2F). And although this minor effect of LPS (compared to saline) was detected in only one of the dietary groups, there was no statistically significant difference between HFD and LFD with relation to IL-10 (Figure 2F).

As a consequence of these diet-induced profile changes, the TNF-α/IL-10 ratio in the LPS-stimulated AT-SVF culture was higher in the HFD group than in the LFD group, whereas there was no intergroup difference in the IL-1β/IL-10 ratio (Figure 2G).

### Alveolar Macrophages

**Figure 3:**
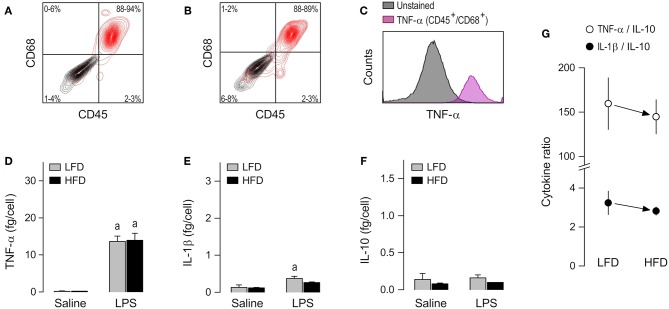
Macrophage abundance and cytokine secretion in alveolar cultures. **(A,B)** Representative flow-cytometry density plots of staining for CD45 (a leukocyte marker) and CD68 (a rat macrophage marker) in alveolar cells from LFD-fed rats **(A)** or HFD-fed rats **(B)**. Stained cells are shown in red; unstained cells are shown in gray. Group results—*n* = 5 culture replicates in **(A)**; *n* = 8 in **(B)**—are shown as 95% confidence intervals within each quadrant. **(C)** Representative histograms showing that LPS-stimulated cells that are CD45^+^/CD68^+^ (macrophages) stain strongly for TNF-α. Because CD45^+^/CD68^−^ were very rare in the alveolar culture, their histograms could not be reliably determined and, consequently, this subpopulation is not shown in **(C)** of this particular figure. For staining intensities of all samples, see Supplemental Table 1. **(D–F)** Effects of diet and *ex-vivo* stimulation with LPS on the secretion of TNF-α **(D)**, IL-1β **(E)**, and IL-10 **(F)** by alveolar macrophages. To aid comparison of this macrophage subpopulation with the others, the scales in these panels are identical to those of the corresponding panels in Figures 2, 4. Data in **(D–F)** are expressed as means ± SEM. Sample size (number of culture replicates) was 6 in the LFD group not stimulated with LPS (saline), 7 in the HFD group not stimulated with LPS (saline), 18 in the LFD group stimulated with LPS, and 16 in the HFD group stimulated with LPS. **(G)** TNF-α/IL-10 and IL-1β/IL-10 ratios (means ± SEM) in the LPS-stimulated alveolar macrophages from the LFD vs. HFD groups. Statistical mark: ^a^significant effect of LPS (compared to saline).

The alveolar cell culture consisted almost exclusively of TNF-producing CD45^+^/CD68^+^ cells (Figures 3A–C and Supplemental Table 1). The cytokine response of alveolar macrophages to LPS was, for the most part, limited to an increase in TNF-α secretion (Figure 3D). IL-10 was never increased by LPS in this macrophage subpopulation (Figure 3F), and even when an increased secretion of IL-1β was detectable, its magnitude was very small (Figure 3E). Most importantly, direct comparisons of the LFD and HFD groups in this macrophage subpopulation did not reveal any significant effect of diet on cytokine secretion (Figures 3D–F) or cytokine ratios (Figure 3G), regardless of whether the alveolar macrophages were stimulated or not with LPS.

### Peritoneal Macrophages

**Figure 4:**
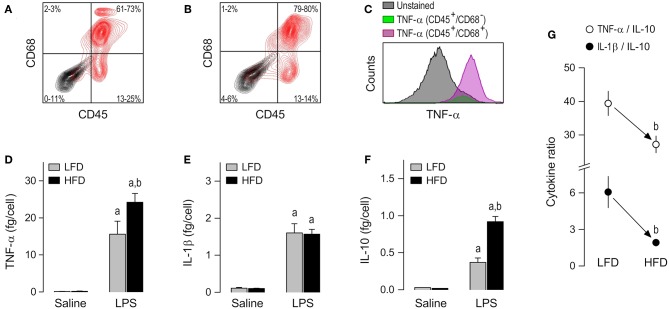
Macrophage abundance and cytokine secretion in peritoneal cultures. **(A,B)** Representative flow-cytometry density plots of staining for CD45 (a leukocyte marker) and CD68 (a rat macrophage marker) in peritoneal cells from LFD-fed rats **(A)** or HFD-fed rats **(B)**. Stained cells are shown in red; unstained cells are shown in gray. Group results—*n* = 5 culture replicates in **(A)**; *n* = 8 in **(B)**—are shown as 95% confidence intervals within each quadrant. **(C)** Representative histograms showing that LPS-stimulated cells that are CD45^+^/CD68^+^ (macrophages) stain more strongly for TNF-α than the other cellular phenotypes present in the peritoneal culture. For staining intensities of all samples, see Supplemental Table 1. **(D–F)** Effects of diet and *ex-vivo* stimulation with LPS on the secretion of TNF-α **(D)**, IL-1β **(E)**, and IL-10 **(F)** by peritoneal macrophages. To aid comparison of this macrophage subpopulation with the others, the scales in these panels are identical to those of the corresponding panels in Figures 2, 3. Data are expressed as means ± SEM. Sample size (number of culture replicates) was 12 in the LFD group not stimulated with LPS (saline), 7 in the HFD group not stimulated with LPS (saline), 16 in the LFD group stimulated with LPS, and 13 in the HFD group stimulated with LPS. **(G)** TNF-α/IL-10 and IL-1β/IL-10 ratios (means ± SEM) in the LPS-stimulated peritoneal macrophages from the LFD vs. HFD groups. Statistical marks: ^a^significant effect of LPS (compared to saline), ^b^significant effect of diet.

The third, and last, subpopulation of resident cells studied was the peritoneal macrophage. CD45^+^/CD68^+^ cells were the most abundant (61–80%) phenotype in peritoneal cultures (Figures 4A,B), as well as the phenotype that stained more strongly for TNF-α following stimulation with LPS (Figure 4C and Supplemental Table 1). As in the case of AT-SVF macrophages, the experiments with peritoneal macrophages uncovered effects of diet on LPS-induced cytokine secretion, but not on basal cytokine secretion. Nevertheless, the response profile of peritoneal macrophages to LPS and its modulation by diet were quite distinct from those of AT-SVF cells. First, the peritoneal macrophages responded to LPS with sizable increases in the secretion of not only TNF-α (Figure 4D), but also IL-1β (Figure 4E), and IL-10 (Figure 4F). Second, peritoneal macrophages of the HFD group displayed enhanced secretion not only of TNF-α (Figure 4D), but also of IL-10 (Figure 4F), while not differing from the LFD group in terms of IL-1β secretion (Figure 4E). Third, and most importantly, owing to a more pronounced enhancement in IL-10 secretion than in TNF-α secretion, peritoneal macrophages from the HFD group exhibited a lower TNF-α/IL-10 ratio than their LFD group counterparts (Figure 4G). Enhanced IL-10 secretion also resulted in a significantly lower IL-1β/IL-10 ratio in the LPS-stimulated peritoneal macrophages of the HFD group (Figure 4G).

### Nuclear Accumulation of NF-κB in AT-SVF vs. Peritoneal Macrophages

**Figure 5:**
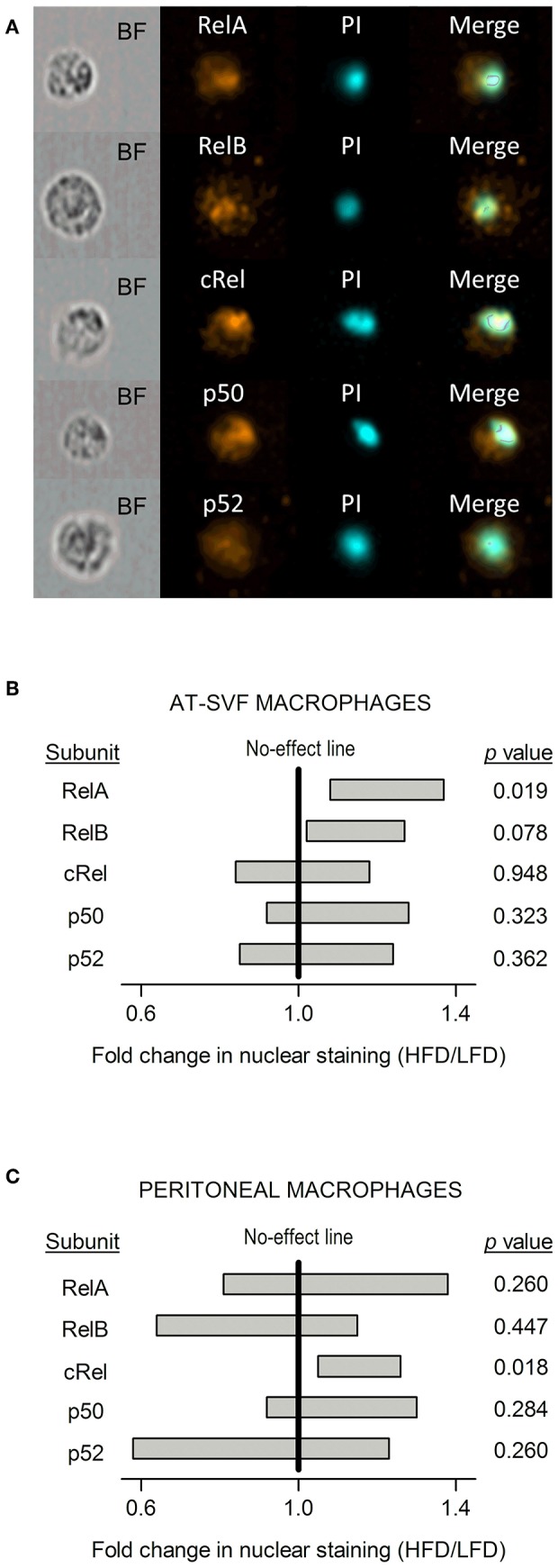
Effects of HFD-induced obesity on the nuclear accumulation of NF-κB subunits in cultures of AT-SVF or peritoneal macrophages stimulated with LPS. **(A)** Representative photos taken by flow-cytometry imaging of cells stained for an NF-κB subunit (RelA, RelB, cRel, p50, or p52; orange pseudocolor) and for the nuclear marker propidium iodide (PI; cyan pseudocolor). BF, bright field. These representative photos are from AT-SVF macrophages; stainings of the same quality were obtained from peritoneal macrophages. **(B)** Effects of HFD-induced obesity on the nuclear localization of NF-κB subunits in AT-SVF macrophages stimulated with LPS. **(C)** Effects of HFD-induced obesity on the nuclear localization of NF-κB subunits in peritoneal macrophages stimulated with LPS. Data in **(B,C)** are shown as 95% confidence intervals (horizontal bars) of the diet-induced fold change in nuclear localization (HFD/LFD). In AT-SVF macrophages, sample sizes (number of culture replicates) were: 8 for RelA; 6 for RelB; 5 for cRel; 6 for p50; and 5 for p52. In peritoneal macrophages, sample sizes were: 6 for RelA; 6 for RelB; 5 for cRel; 7 for p50; and 5 for p52. Values of *p* < 0.05 indicate an effect of diet—fold change significantly different from one.

To evaluate whether the distinct diet-dependent cytokine programs of AT-SVF and peritoneal macrophages could be linked to altered regulation of gene expression by LPS-induced transcription factors, nuclear content of the five known NF-κB subunits (RelA, RelB, cRel, p50, and p52) was assessed by imaging flow cytometry (Figure 5A). This analysis was done in LPS-stimulated cultures, within the CD68^+^ gate. Statistically significant effects of diet were detected for the nuclear accumulation of RelA and cRel, and, most importantly, these effects occurred in a macrophage subpopulation-specific fashion. In the AT-SVF macrophages, nuclear RelA was enhanced in the HFD-induced obesity group in relation to the LFD group (fold change significantly higher than one), while nuclear cRel was not significantly affected (Figure 5B). A trend (*p* = 0.078) toward enhanced nuclear accumulation of RelB was also noticed in the AT-SVF macrophages from obese rats (Figure 5B). On the other hand, in peritoneal macrophages, obesity significantly boosted the nuclear accumulation of cRel, without exerting any effect or trend to an effect on RelA or RelB (Figure 5C).

## Discussion

Although it is becoming increasingly recognized that obesity is a major determinant of outcome in infectious diseases and their most serious complication (sepsis), the interactions among obesity, infectious agents and the immune system are only beginning to be uncovered (2, 25). Previous studies involving assessment of plasma cytokines (26–29) have led to the perception that obesity promotes a global enhancement in the acute pro-inflammatory response to a common PAMP—bacterial LPS. Here, we provide evidence that this matter is more complex than currently envisioned, with obesity exerting dissimilar, tissue-specific effects on subpopulations of resident macrophages.

By culturing freshly excised resident macrophages under identical conditions, our intention was to eliminate their modulation by extrinsic factors that make up the distinct tissue microenvironment of obese vs. lean animals. Such factors include hormones with immunomodulatory actions (17, 30, 31), in addition to cytokines, lipid-derived mediators and gaseous transmitters (32). In the absence of such factors, diet-dependent changes in macrophage biology likely reflect reprogramming of their intrinsic properties. A similar approach was employed in two previous studies involving alveolar macrophages (33, 34), and, like in the present study, no effect of obesity was found on the responsiveness of this macrophage subpopulation to LPS. The present study, however, identified two subpopulations of resident macrophages that had their intrinsic responsiveness to LPS reprogrammed in diet-induced obesity: adipose tissue and peritoneal macrophages.

When stimulated with LPS, all macrophage subpopulations investigated in the present study assumed a pro-inflammatory, M1-like phenotype (35, 36) characterized by TNF-α/IL-10 ratios between 5 and 200. Within this range of M1 polarity, the adipose tissue (AT-SVF) macrophages had the lowest TNF-α/IL-10 ratios, and switched their cytokine response to LPS to a more pro-inflammatory profile in HFD-induced obesity. In contrast, peritoneal macrophages displayed TNF-α/IL-10 ratios that were intermediary to AT-SVF and alveolar macrophages, and, unexpectedly, assumed a less pro-inflammatory cytokine response to LPS in obesity. Unlike TNF-α and IL-10 responses, the IL-1β response to LPS was not significantly affected by diet in any of the macrophage subpopulations investigated, even though sample sizes were quite large in some cases. Hence, reprogramming of macrophage responsiveness to LPS in obesity does not seem to rely on inflammasome-dependent mechanisms, at least as far as IL-1β secretion and the time frame covered by the present study (4 h post-LPS) are concerned. Experiments involving other inflammasome-dependent cytokines (e.g., IL-18) and/or longer periods of macrophage stimulation might reveal that this is an oversimplified picture.

The rate-limiting event for secretion of TNF-α and IL-10 is the upregulation of their gene expression by PAMP-activated transcription factors, chief among which is the translocation of NF-κB to the nucleus (37–40). In this scenario, the present study revealed diet-dependent alterations in the nuclear accumulation of NF-κB subunits in LPS-stimulated macrophages: whereas the RelA subunit had its nuclear content significantly modulated by diet in AT-SVF macrophages, it was the cRel subunit that had its nuclear content impacted by diet in peritoneal macrophages. Considering that NF-κB functions as a dimer (41, 42), the fact that a single subunit was affected in each macrophage subpopulation could be seen as evidence of subunit homodimerization. But although homodimers of RelA or cRel exist (43, 44), it is also possible that, due to promiscuity in heterodimer formation, changes in subunits not exclusively bound to RelA or cRel may have gone undetected in our independent subunit analysis by imaging flow cytometry. For example, the subunit p50 can form heterodimers with all other NF-κB subunits (41), so that changes in the translocation of a single p50-containing heterodimer may not be sufficient to produce detectable changes in the overall nuclear level of this subunit. In future studies, electrophoretic mobility shift assays or chromatin immunoprecipitation assays may be used to specifically define which NF-κB dimers bind to DNA in subpopulations of LPS-stimulated resident macrophages. These studies will be challenging, though, since the assays involved usually require larger quantities of sample than the flow cytometry method employed in the present study.

An important implication of the present study is that it opens avenues for investigations on how subpopulations of resident macrophages could help or harm an obese host during an infection. As pointed out in the *Introduction*, the link between obesity and infectious diseases is currently controversial, with both favorable and unfavorable outcomes having been reported. Perhaps this controversy may have something to do with the relative contributions of distinct subpopulations of macrophages to infections at different primary sites. Accordingly, while obese mice fare worse than lean mice in pulmonary viral or bacterial infections (45, 46), they seem to fare better in bacterial peritonitis (5). Therefore, it is not unreasonable to speculate that by switching the cytokine program of LPS-stimulated peritoneal macrophages to a less pro-inflammatory profile, obesity may aid the host in cases of peritoneal infections. This hypothesis is consistent with the inverse relationship between ROS-dependent microbial killing and the production of pro-inflammatory cytokines (47–49), as well as with a previous investigation in which the phagocytic and microbicidal activities of peritoneal macrophages were enhanced rather than suppressed in diet-induced obesity (50). This hypothesis is also in line with the broader notion that excessive M1 polarization may be counterproductive in the fight against infections (35).

As a final consideration, it should be pointed out that although the approach employed herein was adequate to reveal effects of obesity on LPS-induced cytokine responses, it did not reveal any effect on the low levels of cytokines secreted by cultured macrophages under basal conditions (without LPS stimulation). A priori, this can strike one as a surprise in view of classical reports of AT-SVF macrophages switching their resting state from an anti-inflammatory, M2-like profile to a pro-inflammatory, M1-like profile in obesity (11–14). More recent studies, however, have provided compelling evidence that this qualitative switch in the resting state of AT-SVF macrophages is not always present in obesity (51, 52). One should also consider that the approach employed in the present study differs from that employed in the classical studies in four major ways. First, it should be considered that the classical studies have profiled resting AT-SVF macrophages based on altered gene expression, which might not always be translated into altered cytokine secretion. Second, whereas gene expression profiling in the previous studies was done taking tens of genes into consideration, the present study was limited to the analysis of three cytokines of renowned importance for pathogen-induced acute inflammatory responses. Third, whereas profiling in the classical studies traced back to the time the cells were extracted from the different tissue microenvironments of obese vs. lean animals, profiling in the present study was done after several hours of culture under identical conditions. Fourth, whereas the classical studies were conducted in mice, the present study was carried out in rats.

In conclusion, the present study provides evidence that diet-induced obesity reprograms the intrinsic responsiveness of resident macrophages to bacterial LPS in a tissue-dependent manner. As far as early cytokine secretion is concerned, macrophages residing in lung alveoli did not have their responsiveness to LPS reprogrammed in obesity, but macrophages residing in adipose tissue and peritoneum did. What is more, whereas the adipose tissue macrophages switched their LPS responsiveness to a more pro-inflammatory program, peritoneal macrophages switched their responsiveness to a less pro-inflammatory program. These findings open new avenues to understand the impacts of obesity on PAMP-induced immune responses at the level of macrophage subpopulations that display remarkable plasticity.

## Data Availability

The datasets generated for this study are available on request to the corresponding author.

## Ethics Statement

All protocols were approved by the Animal Care and Use Committee at the Institute of Biomedical Sciences of the University of São Paulo.

## Author Contributions

EK, MF, and AS conceived and designed the study. RM and AS provided essential resources for the study. EK, MF, SdSC-M, WT, and LF performed the experiments and processed the data. EK, MF, and AS performed the formal analyses of the data. EK, MF, SdSC-M, RM, and AS interpreted the data. EK, MF, and AS prepared the figures and drafted the manuscript. All authors edited, revised, and approved the final version of the manuscript.

### Conflict of Interest Statement

The authors declare that the research was conducted in the absence of any commercial or financial relationships that could be construed as a potential conflict of interest.
