# Supplementary material for: Site-Specific Reprogramming of Macrophage Responsiveness to Bacterial Lipopolysaccharide in Obesity
Source: Front Immunol. 2019 Jun 28;10:1496. doi: 10.3389/fimmu.2019.01496 (PMC6611339; doi:10.3389/fimmu.2019.01496)
Supplement: Supplementary file 1 [file Data_Sheet_1.PDF]

SUPPLEMENTAL MATERIAL

Site-specific reprogramming of macrophage responsiveness to bacterial lipopolysaccharide in obesity. Komegae EN, Fonseca MT, da Silveira Cruz-Machado S, Turato WM, Filgueiras LR, Markus RP, Steiner AA

Supplemental Table 1. Individual median fluorescence intensities for TNF-α staining in macrophage cultures.

Diet	Culture type	Unstained controls	Subpopulation		
			CD45⁻/CD68⁻	CD45⁺/CD68⁻	CD45⁺/CD68⁺
LFD	AT-SVF	199	528	630	1,325
			837	964	1,798
			147	401	769
			788	857	1,434
HFD	AT-SVF	244	487	814	1,680
			376	814	1,567
			351	828	1,689
			384	895	1,741
			499	910	1,748
			502	956	1,798
			387	895	1,784
			409	871	1,699
LFD	Alveolar	916	N/A	N/A	5,838
			N/A	N/A	6,281
HFD	Alveolar	873	N/A	N/A	4,088
			N/A	N/A	3,731
			N/A	N/A	3,364
			N/A	N/A	3,608
			N/A	N/A	4,617
			N/A	N/A	4,479
			N/A	N/A	4,534
			N/A	N/A	4,465
LFD	Peritoneal	379	N/A	1,938	2,299
			N/A	2,785	3,521
HFD	Peritoneal	343	N/A	1,073	1,496
			N/A	1,060	1,514
			N/A	1,001	1,403
			N/A	1,151	1,552
			N/A	1,212	1,629
			N/A	1,277	1,700
			N/A	1,316	1,796
			N/A	1,242	1,639

LFD, low-fat diet; HFD, high-fat diet; AT-SVF, adipose tissue stromal vascular fraction.

N/A, not applicable — low cell counts in the gate precluded reliable determination of median fluorescence intensity.
